# Comparative analysis of similarity measurements in miRNAs with applications to miRNA-disease association predictions

**DOI:** 10.1186/s12859-020-3515-9

**Published:** 2020-05-04

**Authors:** Hailin Chen, Ruiyu Guo, Guanghui Li, Wei Zhang, Zuping Zhang

**Affiliations:** 1 School of Software, East China Jiaotong University, Nanchang, 330013 China; 2 School of Information Engineering, East China Jiaotong University, Nanchang, 330013 China; 3 School of Science, East China Jiaotong University, Nanchang, 330013 China; 4 School of Computer Science and Engineering, Central South University, Changsha, 410083 China

**Keywords:** miRNA-disease association, Similarity measurement, Performance evaluation

## Abstract

**Background:**

As regulators of gene expression, microRNAs (miRNAs) are increasingly recognized as critical biomarkers of human diseases. Till now, a series of computational methods have been proposed to predict new miRNA-disease associations based on similarity measurements. Different categories of features in miRNAs are applied in these methods for miRNA-miRNA similarity calculation. Benchmarking tests on these miRNA similarity measures are warranted to assess their effectiveness and robustness.

**Results:**

In this study, 5 categories of features, i.e. miRNA sequences, miRNA expression profiles in cell-lines, miRNA expression profiles in tissues, gene ontology (GO) annotations of miRNA target genes and Medical Subject Heading (MeSH) terms of miRNA-associated diseases, are collected and similarity values between miRNAs are quantified based on these feature spaces, respectively. We systematically compare the 5 similarities from multi-statistical views.

Furthermore, we adopt a rule-based inference method to test their performance on miRNA-disease association predictions with the similarity measurements. Comprehensive comparison is made based on leave-one-out cross-validations and a case study. Experimental results demonstrate that the similarity measurement using MeSH terms performs best among the 5 measurements. It should be noted that the other 4 measurements can also achieve reliable prediction performance. The best-performed similarity measurement is used for new miRNA-disease association predictions and the inferred results are released for further biomedical screening.

**Conclusions:**

Our study suggests that all the 5 features, even though some are restricted by data availability, are useful information for inferring novel miRNA-disease associations. However, biased prediction results might be produced in GO- and MeSH-based similarity measurements due to incomplete feature spaces. Similarity fusion may help produce more reliable prediction results. We expect that future studies will provide more detailed information into the 5 feature spaces and widen our understanding about disease pathogenesis.

## Background

miRNAs are a large family of endogenous non-coding RNA molecules with approximately 22 nucleotides in length. They regulate the expression of their targeted messenger RNAs (mRNAs) through base pairing for cleavage or translational repression [1, 2]. To data, a great number of studies have identified that miRNAs are involved in various crucial biological processes, such as tissue development, cell proliferation and cell death. For example, Sabirzhanov et al. [3] found that a miRNA entitled *miR-711* played a role in neuronal cell death by directly targeting the mRNA *Ang-1* and decreasing its expression. Therefore, the dysfunctions of miRNAs would be associated with the pathogenesis and progression of a spectrum of human complex diseases (e.g. leukemia and cancers) [4]. In addition, as regulators of multiple genes, miRNAs harbor particular therapeutic effects [5–7] and research efforts [8–10] have demonstrated that miRNAs have the potential to become drug targets for disease treatments.

Given the importance of miRNAs in human health, several databases [4, 11, 12], which record associations between miRNAs and diseases by text-mining the published literature, have been launched as valuable resources for public use. In order to reduce the cost of biomedical experiments, computational methods [13–36] have been continuously presented to predict novel miRNA-disease associations for further experimental screening. The hypothesis behind these algorithms is that miRNAs with similar functions would be associated with diseases with similar phenotypes, and vice versa [37]. For instance, Chen et al. [13] adopted random walks on a miRNA-miRNA functional similarity network [38] to prioritize potential miRNAs for diseases of interest. Based on matched miRNA and mRNA expression profiles, Xu et al. [39] systematically identified the most promising miRNAs for cancers through inferred similarity values between miRNA target genes and known disease genes. To improve prediction accuracy, Liu et al. [22] integrated multiple data sources (e.g. miRNA-target gene associations and miRNA-lncRNA associations) for similarity calculation and implemented random walks on miRNA-disease heterogeneous networks for novel miRNA-disease association predictions. More recently, Yang et al. [40] computed similarity between miRNAs using a new GO semantic similarity metric based on miRNA target genes, and proposed a modified correlation model to infer miRNA-disease associations.

These computational approaches constitute an essential alternative to experimental assays. For these methods, it is no doubt similarity measurements are a key factor in determining their prediction accuracy. As to miRNA-miRNA similarity calculation, diverse categories of features, including miRNA sequences, expression profiles of miRNAs and GO of miRNA target genes, have been employed in these methods. However, as far as we know, there are few efforts made in comprehensively comparing the effects of miRNA similarity values, obtained from different features, on inferring novel miRNA-disease associations.

In this study, we first download 5 types of features from miRNAs and calculate their pairwise similarity values based on these feature spaces. Statistical tests are made on the datasets to compare properties of the similarity measurements. Then, we apply the similarity measurements for miRNA-disease association predictions using a popular rule-based inference method. Leave-one-out cross-validations and a case study are implemented for performance assessment and comparison. The best-performed similarity dataset is further used for new miRNA-disease association predictions. Finally, we comprehensively discuss the impacts of the 5 features on similarity calculation and miRNA-disease association predictions from multiple viewpoints, which we expect would provide a reference for biologists when investigating the functions of miRNAs.

## Results

### Overview of the 5 types of similarity measurements

In this study, we collect 5 types of features in miRNAs for pairwise similarity measurements (see Methods). For fair comparison, we use the latest information in each type for similarity calculation.

**Fig. 1:**
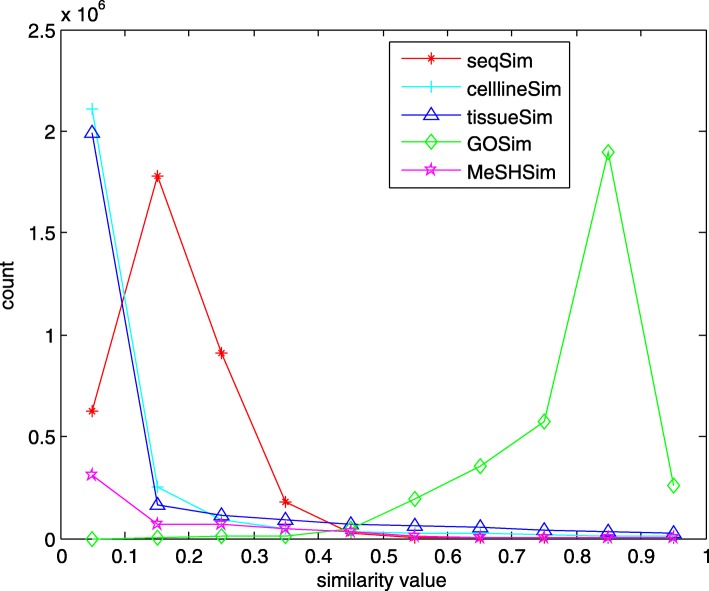
The distributions of pairwise similarity values of miRNAs in the 5 datasets

**Fig. 2:**
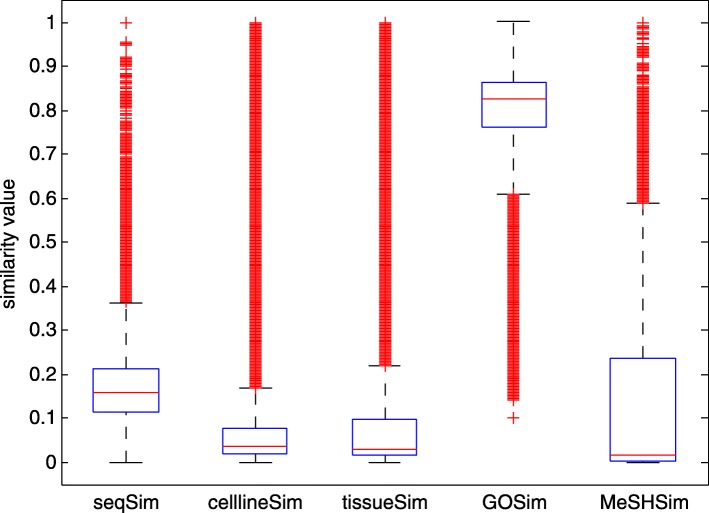
Boxplot of similarity values of miRNAs in the 5 datasets

Table 1 provides a whole view of the information in the 5 datasets. Because of difference in feature availability, the numbers of miRNAs in the 5 datasets vary much with the highest number 2656 in *seqSim* and the lowest 1044 in *MeSHSim*, of which 205 miRNAs are commonly-owned. The distributions of pairwise similarity values in the 5 datasets can be seen in Fig. 1. We further use a boxplot (Fig. 2) to represent the similarity values in the 5 datasets. Four types of statistical results (mean value, standard deviation, skewness and kurtosis) of similarity values in the 5 datasets are calculated and we list them in Table 2.

**Table 1:** An overview of the 5 types of similarity measurements for miRNAs

Name of similarity matrix	No. of miRNAs	Feature for similarity calculation	Year of data published
*seqSim*	2656	miRNA sequences	2018
*celllineSim*	2295	expression profiles in cell lines	2017
*tissueSim*	2300	expression profiles in tissues	2017
*GOSim*	2588	GO of miRNA target genes	2018
*MeSHSim*	1044	MeSH terms of miRNA-associated diseases	2019

**Table 2:** Four types of statistical results of similarity values in the 5 datasets

	mean value	standard deviation	skewness	kurtosis
*seqSim*	0.1682	0.0783	0.8617	5.5952
*celllineSim*	0.0860	0.1436	3.4613	16.1542
*tissueSim*	0.1230	0.2063	2.3258	7.7156
*GOSim*	0.7925	0.1101	−1.5978	6.0010
*MeSHSim*	0.1301	0.1690	1.3665	4.8867

Similarly, we show the distributions of similarity values for the 205 common miRNAs in the 5 datasets in Additional file 1. We also apply a boxplot (Additional file 2) to illustrate similarity values for the 205 common miRNAs. Mean values, standard deviation, skewness and kurtosis for the 205 miRNAs are available at Additional file 3. We discover from the statistical analyses that for each dataset the distributions of similarity values of the whole miRNAs can be well represented by those of the 205 common miRNAs.

### Prediction performance evaluation of the whole miRNAs in each of the 5 datasets

To compare the prediction performance, we first conduct leave-one-out cross-validations for the whole miRNAs in each of the 5 similarity measurements. As shown in Table 3, *MeSHSim* receives the highest average values of ROC-AUC and PR-AUC and performs best in the 5 datasets. The average ROC-AUC value for *MeSHSim* is 0.0389, 0.0394, 0.0406 and 0.0430 higher than these for the other 4 datasets, respectively. Meanwhile, the average PR-AUC value for *MeSHSim* increases by 0.0204, 0.0123, 0.0114 and 0.0265 compared with these for the other 4 datasets, respectively. Note that the other 4 similarity measurements also receive reliable prediction performance.

**Table 3:** Comparison of average values of ROC-AUC and PR-AUC received based on HMDD V3.2 for the whole miRNAs in each of the 5 similarity datasets by leave-one-out cross-validations

	Average ROC-AUC value	Average PR-AUC value
*seqSim*	0.8880	0.2291
*celllineSim*	0.8875	0.2372
*tissueSim*	0.8863	0.2381
*GOSim*	0.8839	0.2230
*MeSHSim*	**0.9269**	**0.2495**

Note:The bold value indicated the highest one in each column

In addition, we implement paired *t*-tests to measure whether the ROC-AUC values and PR-AUC values obtained by *MeSHSim* across the whole miRNAs are significantly higher than these in the other 4 datasets. The calculated *p*-values are available at Table 4. The statistical results demonstrate *MeSHSim* can mostly achieve significantly better performance than all the other 4 measurements at the significance level 0.05.

**Table 4:** Pairwise comparison with paired *t*-tests on the performance results obtained by *MeSHSim* and the other 4 measurements

	*seqSim*	*celllineSim*	*tissueSim*	*GOSim*
*p*-value between *MeSHSim* and another similarity measurement based on ROC-AUC values	1.90783E-20	6.63463E-19	4.96762E-19	4.63222E-23
*p*-value between *MeSHSim* and another similarity measurement based on PR-AUC values	0.023617	0.185257	0.221117	0.00308

**Fig. 3:**
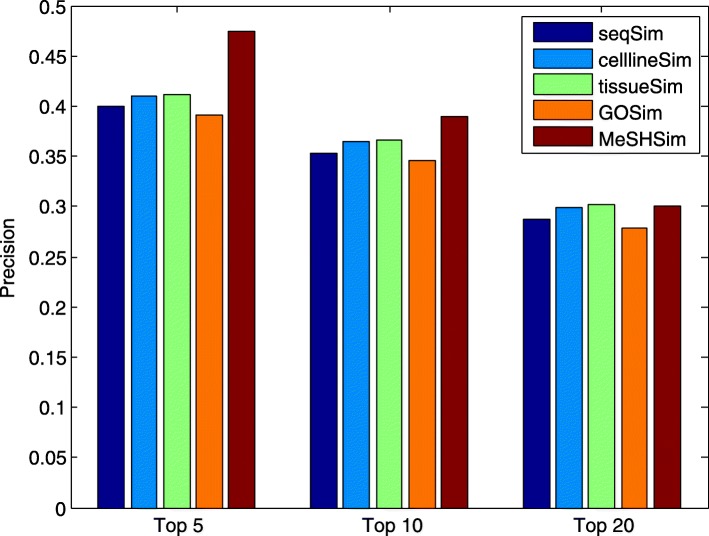
Comparison of average *PRE* values in the top-*k* predictions for the whole miRNAs in each of the 5 datasets by leave-one-out cross-validations based on HMDD V3.2

**Fig. 4:**
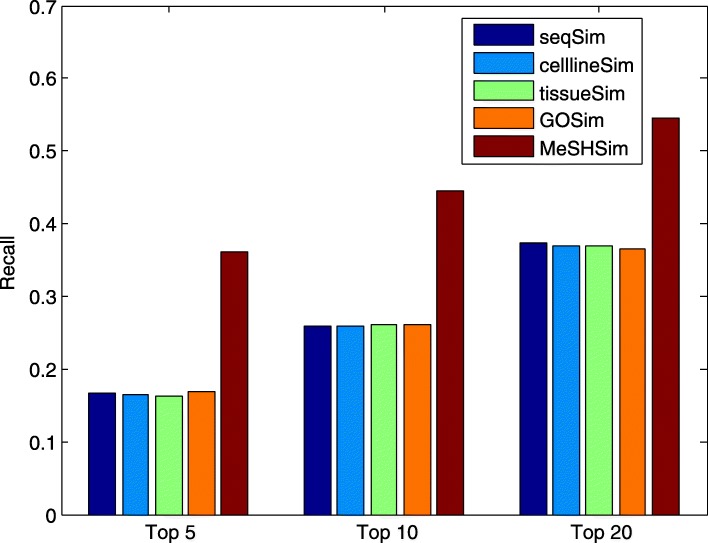
Comparison of average *REC* values in the top-*k* predictions for the whole miRNAs in each of the 5 datasets by leave-one-out cross-validations based on HMDD V3.2

Higher precision and recall values within the top *k* ranking list indicate more positive testing samples (real miRNA-disease associations in our study) are successfully predicted. The average precision and recall values across the whole miRNAs in the 5 datasets within the top *k* candidates are illustrated in Fig. 3 and Fig. 4, respectively. The two figures demonstrate that *MeSHSim* consistently outperforms the other 4 measurements at different *k* cutoffs.

### Prediction performance evaluation of the 205 common miRNAs in each of the 5 datasets

Considering the numbers of miRNAs in each of the 5 similarity datasets are different, we further choose the 205 common miRNAs in the 5 datasets to carry out leave-one-out cross-validation experiments to test their prediction performance.

As shown in Table 5, *MeSHSim* receives the highest average values of ROC-AUC and PR-AUC and performs best in the 5 datasets. The average ROC-AUC value for *MeSHSim* is 0.0267, 0.0363, 0.0372 and 0.0296 higher than these for the other 4 datasets, respectively. Meanwhile, the average PR-AUC value for *MeSHSim* increases by 0.0536, 0.0729, 0.0714 and 0.0606 compared with these for the other 4 datasets, respectively. Table 5 also suggests that the other 4 similarity measurements are able to achieve reliable prediction performance.

**Table 5:** Comparison of average values of ROC-AUC and PR-AUC received based on HMDD V3.2 for the 205 common miRNAs in the 5 similarity datasets by leave-one-out cross-validations

	Average ROC-AUC value	Average PR-AUC value
*seqSim*	0.9114	0.1366
*celllineSim*	0.9018	0.1173
*tissueSim*	0.9009	0.1188
*GOSim*	0.9085	0.1296
*MeSHSim*	**0.9381**	**0.1902**

Note:The bold value indicated the highest one in each column

Paired *t*-tests are implemented to measure whether the ROC-AUC values and PR-AUC values obtained by *MeSHSim* across the 205 common miRNAs are significantly higher than these in the other 4 datasets. The calculated *p*-values are available at Table 6, and statistical results demonstrate *MeSHSim* achieves significantly better performance than all the other 4 measurements at the significance level 0.05.

**Table 6:** Pairwise comparison with paired *t*-tests on the performance results obtained by *MeSHSim* and the other 4 measurements across the 205 common miRNAs

	*seqSim*	*celllineSim*	*tissueSim*	*GOSim*
*p*-value between *MeSHSim* and another similarity measurement based on ROC-AUC values	0.002349188	6.66612E-05	4.42713E-05	0.000785147
*p*-value between *MeSHSim* and another similarity measurement based on PR-AUC values	0.000938522	3.71868E-06	6.19924E-06	0.000176833

**Fig. 5:**
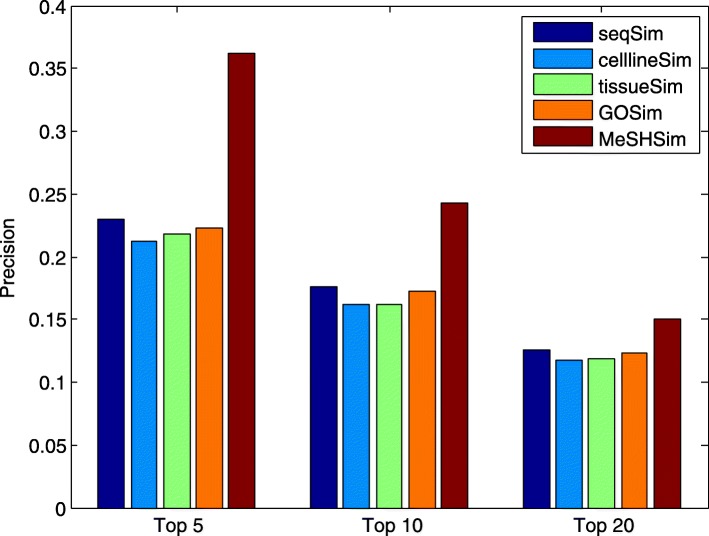
Comparison of average *PRE* values in the top-*k* predictions for the 205 common miRNAs in the 5 datasets by leave-one-out cross-validations based on HMDD V3.2

**Fig. 6:**
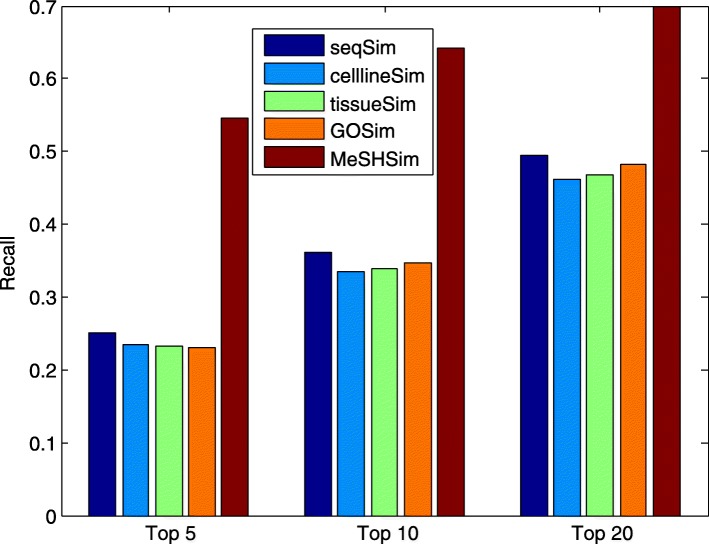
Comparison of average *REC* values in the top-*k* predictions for the 205 common miRNAs in the 5 datasets by leave-one-out cross-validations based on HMDD V3.2

The average precision and recall values across the 205 common miRNAs in the 5 datasets within the top *k* candidates are illustrated in Fig. 5 and Fig. 6, respectively. We can conclude from the 2 figures that *MeSHSim* consistently outperforms the other 4 measurements at various *k* cutoffs.

### A case study

To further compare their abilities to predict potential disease candidates for miRNAs in the 5 datasets, we conduct a case study on *hsa-mir-2861*. The whole 894 disease candidates in the benchmarking dataset are ranked according to our method. We choose the top *k* (*k* = 10, 20, 40, 60, 80 and 100) predicted results for confirmation. We list the numbers of verified results in Table 7, which indicates the superiority of *MeSHSim* in screening the most predicted miRNA-disease associations.

**Table 7:** Confirmed numbers of the top-*k* predicted results of *hsa-mir-2861* in the 5 datasets

	Top 10	Top 20	Top 40	Top 60	Top 80	Top 100
number of confirmed predictions (*seqSim*)	0	0	1	3	4	4
number of confirmed predictions (*celllineSim*)	0	1	1	2	4	4
number of confirmed predictions (*tissueSim*)	0	0	1	2	4	4
number of confirmed predictions (*GOSim*)	0	1	1	2	4	4
number of confirmed predictions (*MeSHSim*)	0	1	3	3	5	5

### Predictions of new miRNA-disease associations

After extensive comparison, we choose the best-performed similarity measurement *MeSHSim* to conduct comprehensive predictions of unknown associations between miRNAs and diseases. Experimentally verified miRNA-disease associations are downloaded from HMDD V3.2. In this inference proceeding, we train the method MBSI (see Method) with all known associations. We rank the non-interacting pairs according to their scores derived from Eq. (1) and extract the top 10 predicted results for each miRNA. The list of predicted associations can be seen in Additional file 4.

## Discussion

In this study, 5 types of features are applied for miRNA similarity calculation. From the viewpoint of data sources, miRNA sequences are the most available, which is confirmed by the numbers of miRNAs in Table 1. As to miRNA expressions, accumulating data are available thanks to biomedical advance. However, it is known that quantitative values of miRNA expressions are affected by factors like library preparation protocols and adapter trimming steps. Therefore, robust pipelines to measure the expression values are well needed. Regarding *GOSim*, functional annotations for miRNAs are scarce in public databases and predicted miRNA target genes are integrated in Reference [40] for similarity calculation. False positive rate of predicted target genes would affect the similarity results and final prediction performance. For *MeSHSim*, it quantifies miRNA functional similarity based on MeSH terms derived from existing miRNA-associated diseases. The number of miRNAs in this dataset would therefore be greatly constrained. Because of incomplete data of experimentally supported miRNA-disease associations, the calculated similarity values in *MeSHSim* may be biased.

Experimental results demonstrate that *MeSHSim* performs best and the other 4 similarity measurements can also achieve stable and reliable prediction abilities. This can be explained with two biological facts, i.e. miRNAs target mRNAs through base pairing and a change in the expression level of a particular miRNA would lead to severe pathological conditions. Therefore, we expect that seamless integration of the 5 kinds of features for similarity measurements would produce more reliable prediction results.

For algorithms to infer miRNA-disease associations, the *cold-start* problem, in which associated diseases need to be predicted for a totally new miRNA, is a challenge that needs to be properly addressed. Strictly speaking, the similarity values in *MeSHSim* should be re-calculated before each round of cross validation is implemented in our study. As these values are computed based on known miRNA-disease associations, algorithms using *MeSHSim* for predictions suffer from the *cold-start* problem. Compared with *MeSHSim*, the other 4 similarity measurements do not encounter such challenge.

We focus only on the impact of miRNA similarity on miRNA-disease association predictions in this study. It is worthy pointing out that disease similarity is also vital for these similarity-based methods to improve their prediction performance, which is a further research topic.

## Conclusions

Pairwise miRNA similarity measurement is an important step for miRNA-disease association predictions. In this study, we collect 5 feature spaces in miRNAs for similarity calculation and apply the similarity values to miRNA-disease association predictions. We comprehensively compare the statistical properties of the similarity values and systematically evaluate their inference performance on one independent benchmarking dataset. Although satisfied experimental results are received in all the 5 datasets, researchers should be cautious of the potential bias caused by some similarity measurements. Approaches allowing similarity fusion are in need for achieving more reliable prediction results.

## Methods

### Data preparation

We exploit 5 widely-used features for miRNA-miRNA similarity measurements. All similarity measures are symmetrically normalized to be in the range of (0, 1). The miRNA-miRNA similarity measures are as follows.

1. *Sequence-based similarity between miRNAs*: We download nucleotide sequences of miRNAs from the latest version of *miRBase* (http://www.mirbase.org/) [41]. The fasta format sequences of 2656 mature miRNAs in *Homo sapiens* in the database are kept and the sequences of miRNAs in other species are removed. The sequence similarity between two miRNAs is computed using *needleall* (http://www.bioinformatics.nl/cgi-bin/emboss/needleall). The parameters for this tool are set according to default values (Matrix file = EDNAfull, Gap opening penalty = 10, Gap extension penalty = 0.5). We refer to the 2656 × 2656 sequence similarity matrix as *seqSim*.
2. *Expression-profile-in-cell-line-based similarity between miRNAs*: We download expression profiles of miRNAs in 24 different types of cell-lines from *miRmine* (http://guanlab.ccmb.med.umich.edu/mirmine/) [42]. After merging miRNAs with the same name and deleting miRNAs with whole expression values of 0, we obtain 2295 mature miRNAs. Absolute values of Pearson correlation coefficient (PCC) between the expression profiles are computed as the measurement of similarity for the miRNAs. We refer to the 2295 × 2295 expression similarity matrix as *celllineSim*.
3. *Expression-profile-in-tissue-based similarity between miRNAs*: We download expression profiles of miRNAs in 16 different types of human tissues and bio fluids from *miRmine* (http://guanlab.ccmb.med.umich.edu/mirmine/) [42]. We take the same data processing steps as these in *celllineSim* and obtain 2300 mature miRNAs. We refer to the 2300 × 2300 expression similarity matrix as *tissueSim*.
4. *GO-of-target-gene-based similarity between miRNAs*: Recently, Yang et al. [40] developed a method entitled MiRGOFS to measure the functional similarity for 2588 miRNAs based on GO annotations of their target genes. We download the similarity results from their study. To normalize the raw data, we divide the value of each element before the diagonal one in each row (and column) by the value of the diagonal element and obtain a symmetric similarity matrix. Note that the normalized similarity matrix in Reference [40] was unsymmetric. We refer to the 2588 × 2588 similarity matrix as *GOSim*.
5. *MeSH-term-of-disease-based similarity between miRNAs*: In 2010, Wang et al. [38] presented a method MISIM to infer pairwise functional similarity for miRNAs based on MeSH terms of miRNA-associated diseases. More recently, an improved and updated version of MISIM (MISIM V2.0 [43]) was released. We download the similarity values of 1044 miRNAs from MISIM V2.0 (http://www.lirmed.com/misim/) and refer to the 1044 × 1044 similarity matrix as *MeSHSim*.

### miRNA-disease association discovering

We adopt one popular rule-based inference method, miRNA-based similarity inference (MBSI) [15], to discover miRNA-disease associations with the similarities obtained from the above section.

**Fig. 7:**
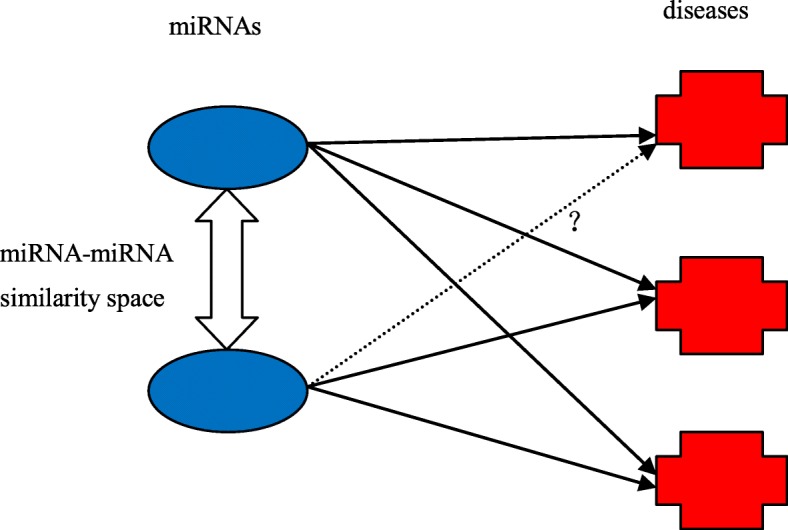
The principle behind new miRNA-disease association predictions. If a miRNA with unknown interaction profile shares a similar property with another miRNA with known interaction profile property, the former may also share the same interaction profile with the latter

We postulate in MBSI if a miRNA is implicated in a disease, similar miRNAs might also be associated with the disease (see Fig. 7). For a pair of miRNA-disease association (*m*_*i*_, *d*_*j*_), the inference score of the pair is calculated as,

1 $$ score\left({m}_i,{d}_j\right)=\frac{\sum_{l=1,l\ne i}^n Sim\left({m}_i,{m}_l\right){a}_{lj}}{\sum_{l=1,l\ne i}^n Sim\left({m}_i,{m}_l\right)} $$

where *m*_*i*_ and *d*_*j*_ denote miRNA *i* and disease *j*, *Sim*(*m*_*i*_, *m*_*l*_) is the similarity value between *m*_*i*_ and *m*_*l*_, and *a*_*lj*_ =1if there is an existing association between *m*_*l*_ and *d*_*j*_, otherwise *a*_*lj*_ =0. A higher score received from Eq. (1) indicates more confidence in a predicted association.

### Validation and evaluation metrics

We obtain a benchmarking dataset from HMDD V3.2 which contained experimentally supported miRNA-disease associations. This gold-standard dataset is regarded as true positive samples and is used for performance test.

We implement leave-one-out cross-validations to evaluate the prediction performance. Specifically, each miRNA is taken out once for testing and the remaining miRNAs for training. For each testing miRNA, all its association information is removed and the predicted scores for its associations with diseases are derived from Eq. (1). We rank the entire disease set for the testing miRNA according to the scores.

For each testing miRNA, we take the known miRNA-disease associations as positive instances. For each specific ranking threshold, if the score of a predicted miRNA-disease association is above the threshold, it is considered as a true positive. Otherwise, it is taken as a false positive. True positive rate (*TPR*), false positive rate (*FPR*), precision (*PRE*) and recall (*REC*) are calculated as follows by varying thresholds to plot ROC and PR curves,

2 $$ TPR=\frac{TP}{TP+ FN} $$

3 $$ TPR=\frac{TP}{TP+ FN} $$

4 $$ PRE=\frac{TP}{TP+ FP} $$

5 $$ REC=\frac{TP}{TP+ FN} $$

where *TP* and *TN* are the numbers of correctly predicted positive and negative samples. *FP* and *FN* are the numbers of misidentified positive and negative samples. We use values of area under the ROC and PR curves (*AUC*) to demonstrate the prediction ability. We also measure the *PRE* and *REC* within the top 5, top 10 and top 20 candidates in the ranking list, because biologists are more interested in the top predictions.

## Supplementary information

**Additional file 1.** The distributions of pairwise similarity values of the 205 common miRNAs in the 5 datasets.

**Additional file 2.** Boxplot of similarity values of the 205 common miRNAs in the 5 datasets.

**Additional file 3.** Four types of statistical results of similarity values of the 205 common miRNAs in the 5 datasets.

**Additional file 4. **The top 10 predicted results for miRNAs in *MeSHSim.*

## Data Availability

The datasets used and/or analysed during the current study are available from the corresponding author on reasonable request.

## Abbreviations

miRNAs: microRNAs
mRNAs: Messenger RNAs
GO: Gene ontology
PCC: Pearson correlation coefficient
MeSH: Medical Subject Headings
MBSI: miRNA-based similarity inference
