# Supplementary figures and images for: Comparative analysis of similarity measurements in miRNAs with applications to miRNA-disease association predictions
Source: BMC Bioinformatics. 2020 May 4;21:176. doi: 10.1186/s12859-020-3515-9 (PMC7199309; doi:10.1186/s12859-020-3515-9)

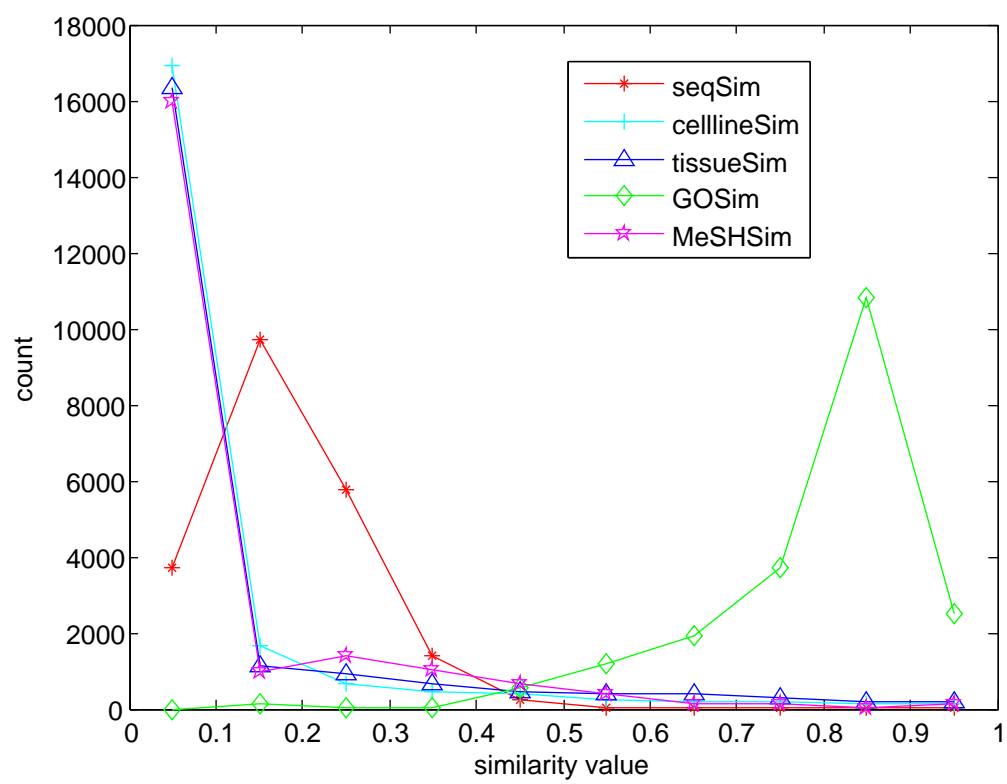

Supplement: Supplementary file 1 — Additional file 1. The distributions of pairwise similarity values of the 205 common miRNAs in the 5 datasets. [file 12859_2020_3515_MOESM1_ESM.pdf]

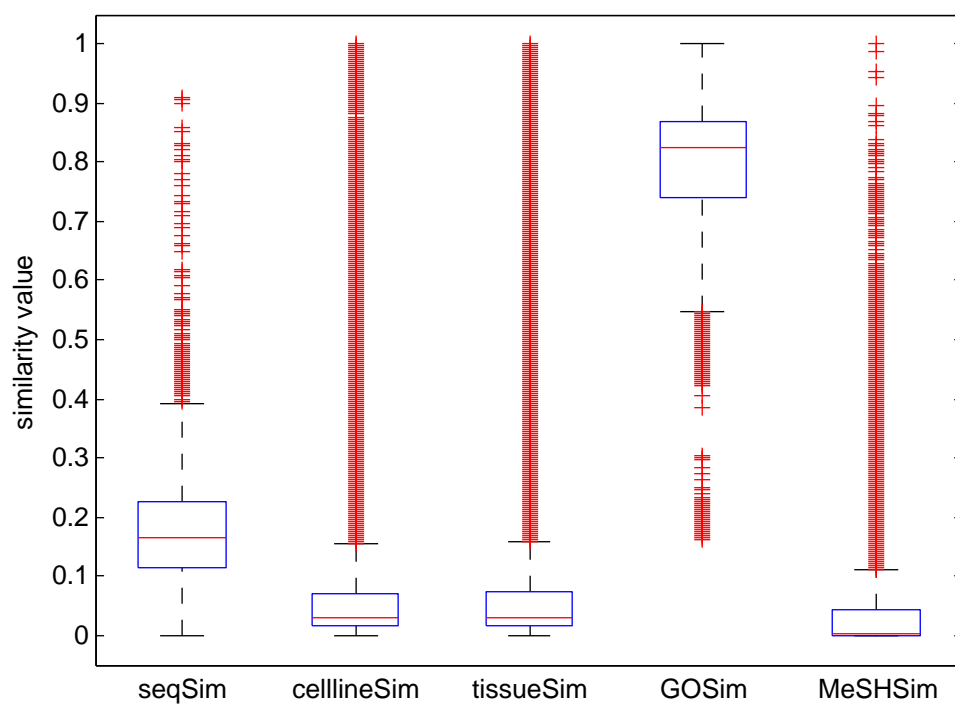

Supplement: Supplementary file 2 — Additional file 2. Boxplot of similarity values of the 205 common miRNAs in the 5 datasets. [file 12859_2020_3515_MOESM2_ESM.pdf]
