# Supplementary material for: Comparative analysis of similarity measurements in miRNAs with applications to miRNA-disease association predictions
Source: BMC Bioinformatics. 2020 May 4;21:176. doi: 10.1186/s12859-020-3515-9 (PMC7199309; doi:10.1186/s12859-020-3515-9)
Supplement: Supplementary file 3 — Additional file 3. Four types of statistical results of similarity values of the 205 common miRNAs in the 5 datasets. [file 12859_2020_3515_MOESM3_ESM.docx]

## Additional file 3 - Four types of statistical results of similarity values of the 205 common miRNAs in the 5 datasets

|  | mean value | standard deviation | skewness | kurtosis |
| --- | --- | --- | --- | --- |
| *seqSim* | 0.1752 | 0.0883 | 1.2327 | 8.1988 |
| *celllineSim* | 0.0882 | 0.1589 | 3.2849 | 14.3377 |
| *tissueSim* | 0.1084 | 0.1892 | 2.5812 | 9.2399 |
| *GOSim* | 0.7888 | 0.1255 | -1.8010 | 7.2746 |
| *MeSHSim* | 0.0864 | 0.1726 | 2.4647 | 9.6349 |
